# Commentary: To slipknot or skip the knot: Preclosure in percutaneous extracorporeal membrane oxygenation cannulation, a misuse of precious time?

**DOI:** 10.1016/j.xjtc.2021.09.036

**Published:** 2021-09-24

**Authors:** Gabriel Georges, Siamak Mohammadi

**Affiliations:** Department of Cardiac Surgery, Quebec Heart and Lung Institute, Quebec City, Quebec, Canada

**Figure:**
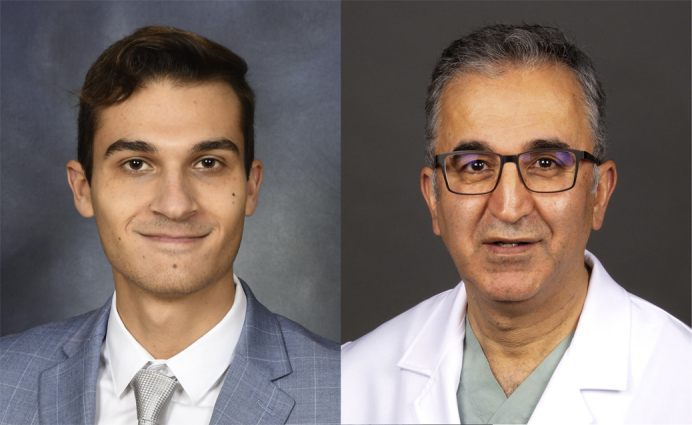
*From left*: Gabriel Georges, MD, and Siamak Mohammadi, MD, FRCSC

**Central Message**

Peripheral percutaneous VA-ECMO decannulation is feasible, has a high success rate, and seems to reduce the number of groin infections.

See Article page 322.

Preclosure techniques of large femoral arteriotomy sites have been shown to be safe and effective in elective procedures such as transcatheter aortic valve replacement.^1^ In this issue of the *Journal*, Chandel and colleagues^2^ evaluate the use of a double-ProGlide preclosure technique at the time of urgent venoarterial extracorporeal membrane oxygenation (VA-ECMO) cannulation. The group retrospectively compared vascular outcomes in 99 consecutive patients managed with femoral VA-ECMO who either received the preclosure technique (N = 51) or underwent standard arterial cutdown at the time of decannulation (N = 48). The authors reported technical success as defined as adequate hemostasis and freedom from arterial stenosis, requiring intervention in 90.2% of patients who had received the Perclose ProGlide system (Abbott Inc, Chicago, Ill). Patients in the preclosure group had a lower rate of limb complications (5.9 vs 25%; *P* = .011), mostly driven by a lower incidence of infection at cannulation site (2 vs 14.6%; *P* = .028). More bleeding events were also reported in the surgical repair group (5.9 vs 22.9%; *P* = .02), although there was a trend toward greater international normalized ratio values at the time of cannulation (1.7 vs 1.4; *P* = .066) and more patients in the surgical group were undergoing cardiopulmonary resuscitation (CPR) at the time of cannulation (29.3 vs 7.8%; *P* = .008). The significant increase in frequency of bleeding events did not persist when patients undergoing CPR during cannulation were excluded from the analysis.

With this study,^2^ Chandel and colleagues contribute to the sparse but growing evidence supporting the safety and feasibility of total percutaneous VA-ECMO support. The novelty of this report is that it represents the largest study in which a preclosure technique was used during urgent femoral VA-ECMO cannulation. Earlier this year, Martin-Tuffreau and colleagues^3^ reported their experience with preclosing for urgent VA-ECMO using the double-ProGlide technique in an observational prospective study extending from 2018 to 2020. However, their cohort consisted of only 20 patients, and decannulation was performed using a crossover technique under angiographic guidance, which requires a hybrid operating room. Their technical success rate (95%) was similar to that of Chandel and colleagues.^2^

The key finding of this article^2^ is that percutaneous VA-ECMO decannulation seems to reduce the number of groin infections. Low rates of cannulation site infections have also been shown using other percutaneous closure techniques following ECMO decannulation.^3^, ^4^, ^5^, ^6^, ^7^ This would suggest that patients predisposed to a greater risk of groin infection (eg, smokers, those with diabetes or obesity) could benefit from a totally percutaneous VA-ECMO management strategy. Nevertheless, this may be modulated by the additional technical challenges related to the deployment of preclosure systems in the patient with obesity.

It is certainly too early to support the authors' standard use of the preclosure technique in all patients considered for VA-ECMO. It should be recognized that the results of this study are derived from a retrospective analysis of a small cohort of patients, especially, given the heterogeneity of patients presenting with cardiogenic shock. In addition, the validity of the findings is diminished by the fact that the use of the preclosure technique was left to the discretion of the surgeon, and thus the inherent selection bias of less critically ill patients who could afford the additional delay before VA-ECMO initiation in the preclosure group. This is well represented by the significantly lower proportion of patients who were undergoing CPR during VA-ECMO cannulation in the preclosure group and may not be accounted for by excluding this specific subset of patients from the analysis. While the rationale for delaying VA-ECMO cannulation in critically ill patients by deploying preclosure devices may be difficult to defend, percutaneous postclosure techniques with the Perclose ProGlide^6^^,^^8^^,^^9^ or MANTA^7^^,^^10^^,^^11^ (Teleflex Inc, Durham, NC) closure devices may prove to be a safe alternative to surgical cut-down in selected patients at high-risk of groin infection.

The authors are to be congratulated for their novel effort in reducing morbidity related to VA-ECMO support. There is growing evidence that total percutaneous cannulation and decannulation VA-ECMO using preclosure devices is safe and feasible, although it remains unclear what populations may benefit the most from this approach.

## References

[1] Griese D.P., Reents W., Diegeler A., Kerber S., Babin-Ebell J. (2013). Simple, effective and safe vascular access site closure with the double-ProGlide preclose technique in 162 patients receiving transfemoral transcatheter aortic valve implantation. Catheter Cardiovasc Interv.

[2] Chandel A., Desai M., Ryan L.P., Clevenger L., Speir A.M., Singh R. (2021). Preclosure technique versus arterial cutdown after percutaneous cannulation for venoarterial extracorporeal membrane oxygenation. J Thorac Cardiovasc Surg Tech.

[3] Martin-Tuffreau A.S., Bagate F., Boukantar M., Saiydoun G., Mangiameli A., Rostain L. (2021). Complete percutaneous angio-guided approach using preclosing for venoarterial extracorporeal membrane oxygenation implantation and explantation in patients with refractory cardiogenic shock or cardiac arrest. Crit Care.

[4] Pellenc Q., Girault A., Roussel A., Aguir S., Cerceau P., Longrois D. (2020). Preclosing of the femoral artery allows total percutaneous venoarterial extracorporeal membrane oxygenation and prevents groin wound infection after lung transplantation. Eur J Cardiothorac Surg.

[5] Xu X., Liu Z., Han P., He M., Xu Y., Yin L. (2019). Feasibility and safety of total percutaneous closure of femoral arterial access sites after veno-arterial extracorporeal membrane oxygenation. Medicine.

[6] Hwang J.W., Yang J.H., Sung K., Song Y.B., Hahn J.Y., Choi J.H. (2016). Percutaneous removal using Perclose ProGlide closure devices versus surgical removal for weaning after percutaneous cannulation for venoarterial extracorporeal membrane oxygenation. J Vasc Surg.

[7] Bemtgen X., Heidt T., Zotzmann V., Rilinger J., Wengenmayer T., Biever P.M. (2020). Venoarterial extracorporeal membrane oxygenation decannulation using the novel Manta vascular closure device. Eur Heart J Acute Cardiovasc Care.

[8] Au S.-Y., Chan K.-S., Fong K.-M., Leung P.W.R., Ng W.-Y.G., Leung K.-H.A. (2020). Bedside decannulation of peripheral VA-ECMO using percutaneous Perclose ProGlide post-close technique. J Emerg Crit Care Med.

[9] Lüsebrink E., Stremmel C., Stark K., Petzold T., Hein-Rothweiler R., Scherer C. (2019). Percutaneous decannulation instead of surgical removal for weaning after venoarterial extracorporeal membrane oxygenation—a crossed Perclose ProGlide closure device technique using a hemostasis valve Y connector. Crit Care Explor.

[10] Montero-Cabezas J.M., van der Meer R.W., van der Kley F., Elzo Kraemer C.V., López Matta J.E., Schalij M.J. (2019). Percutaneous decannulation of femoral venoarterial ECMO cannulas using MANTA vascular closure device. Can J Cardiol.

[11] Hassan M.F., Lawrence M., Lee D., Velazco J., Martin C., Reddy R. (2020). Simplified percutaneous VA ECMO decannulation using the MANTA vascular closure device: initial US experience. J Card Surg.

